# DECTIN-1: A modifier protein in CTLA-4 haploinsufficiency

**DOI:** 10.1126/sciadv.adi9566

**Published:** 2023-12-06

**Authors:** Cynthia Turnbull, Josiah Bones, Maurice Stanley, Arti Medhavy, Hao Wang, Ayla May D. Lorenzo, Jean Cappello, Somasundhari Shanmuganandam, Abhimanu Pandey, Sandali Seneviratne, Grant J Brown, Xiangpeng Meng, David Fulcher, Gaetan Burgio, Si Ming Man, Carmen de Lucas Collantes, Mercedes Gasior, Eduardo López Granados, Pilar Martin, Simon H. Jiang, Matthew C. Cook, Julia I. Ellyard, Vicki Athanasopoulos, Ben Corry, Pablo F. Canete, Carola G. Vinuesa

**Affiliations:** ^1^ John Curtin School of Medical Research, Australian National University, Canberra, Australian Capital Territory, Australia.; ^2^ Research School of Biology, Australian National University, Canberra, Australian Capital Territory, Australia.; ^3^ The Francis Crick Institute, London, UK.; ^4^ Nephrology Department, Hospital Infantil Universitario Niño Jesús, Madrid, Spain.; ^5^ Hematology Department, Hospital Universitario La Paz, Madrid, Spain.; ^6^ Clinical Immunology Department, Hospital Universitario La Paz, Madrid, Spain.; ^7^ Center for Biomedical Network Research on Rare Diseases, Madrid, Spain.; ^8^ Lymphocyte Pathophysiology in Immunodeficiencies Group, La Paz Institute for Health Research, Madrid, Spain.; ^9^ Centro Nacional de Investigaciones Cardiovasculares, Madrid, Spain.; ^10^ Centro de Investigacion Biomedica En Rad, Madrid, Spain.; ^11^ Cambridge Institute for Therapeutic Immunology and Infectious Diseases, University of Cambridge, Cambridge, UK.; ^12^ Frazer Institute, The University of Queensland, Woolloongabba, Queensland, Australia.

## Abstract

Autosomal dominant loss-of-function (LoF) variants in cytotoxic T-lymphocyte associated protein 4 (*CTLA4*) cause immune dysregulation with autoimmunity, immunodeficiency and lymphoproliferation (IDAIL). Incomplete penetrance and variable expressivity are characteristic of IDAIL caused by CTLA-4 haploinsufficiency (CTLA-4h), pointing to a role for genetic modifiers. Here, we describe an IDAIL proband carrying a maternally inherited pathogenic *CTLA4* variant and a paternally inherited rare LoF missense variant in *CLEC7A,* which encodes for the β-glucan pattern recognition receptor DECTIN-1. The *CLEC7A* variant led to a loss of DECTIN-1 dimerization and surface expression. Notably, DECTIN-1 stimulation promoted human and mouse regulatory T cell (T_reg_) differentiation from naïve αβ and γδ T cells, even in the absence of transforming growth factor–β. Consistent with DECTIN-1’s T_reg_-boosting ability, partial DECTIN-1 deficiency exacerbated the T_reg_ defect conferred by CTL4-4h. DECTIN-1/*CLEC7A* emerges as a modifier gene in CTLA-4h, increasing expressivity of *CTLA4* variants and acting in functional epistasis with CTLA-4 to maintain immune homeostasis and tolerance.

## Teaser

DECTIN-1 modifies CTLA-4 deficiency, exacerbating Treg defects.

## INTRODUCTION

Inborn errors of immunity (IEIs) are a heterogeneous group of genetic abnormalities that lead to perturbations of the immune system. While these conditions were traditionally thought to encompass primary immune deficiencies, with susceptibility to infections as a major clinical hallmark, it is now clear that some IEIs exhibit autoimmune features or a combination of both, as seen in immune dysregulation with autoimmunity, immunodeficiency, and lymphoproliferation (IDAIL). Although IDAIL is complex and heterogeneous in both genetic etiology and clinical phenotypes, most patients exhibit T cell hyperactivity. Not surprisingly, disruptions in genes that control T cell immune checkpoints, T cell tolerance, and regulatory T cells (T_regs_), which use a variety of strategies to suppress effector T cells, often lead to immune dysregulation. Indeed, loss-of-function (LoF) variants in genes important for T_reg_ differentiation, function, and maintenance, such as *FOXP3*, cytotoxic T-lymphocyte associated protein 4 (*CTLA4*), LPS Responsive Beige-Like Anchor Protein (*LRBA*), and *CD25,* result in various forms of IDAIL (*1*).

CTLA-4 haploinsufficiency (CTLA-4h) is a particularly severe but highly variable immune disorder characterized by the presence of damaging gene variants in *CTLA4* (*1*–*3*). CTLA-4 is a transmembrane receptor predominantly expressed on T_regs_, which is indispensable for maintaining immune tolerance and homeostasis (*4*). Being structurally similar to the T cell costimulatory molecule CD28, it restrains T cell responses predominantly by binding to and removing the CD28 ligands, CD80 and CD86, from the cell surface of antigen-presenting cells, thus precluding T cells from receiving adequate activation and proliferation signals (*5*, *6*). Unlike homozygous *Ctla4*-deficient mice, which exhibit massive T cell lymphoproliferation, multi-organ damage, and premature death, heterozygous mice remain healthy (*7*). Selective elimination of CTLA-4 in the T_reg_ compartment only delays onset of symptoms and mice eventually succumb to disease (*8*), thus highlighting the importance of T_reg_-expressed CTLA-4 for mediating immune tolerance. In contrast, human *CTLA4* variants typically act in an autosomal dominant manner to cause complex IDAIL similar to that seen in mice (*4*). The presenting clinical manifestations include lymphoproliferation, autoimmune endocrinopathies, autoimmune cytopenias, lymphocytic infiltration of the gastrointestinal system, lungs, and brain, and combined variable immunodeficiency manifestations such as hypogammaglobulinemia and recurrent respiratory infections (*1*–*3*).

Incomplete penetrance and variable expressivity are the norm for CTLA-4h–mediated IDAIL, with 30 to 40% of carriers of pathogenic *CTLA4* variants showing no clinical symptoms (*1*–*3*). This is despite both affected and unaffected mutation carriers showing comparable defects in CTLA-4 expression and function in vitro (*1*–*3*). Several studies have ruled out the contribution of genetic and environmental factors known to explain other incompletely penetrant IEIs such as biallelic genetic deficiencies of *CTLA4* (e.g., frameshifts and deletions), somatic mutations, or past viral infections (*1*, *2*, *9*). Given that phenotypic manifestations of many gene variants depend on interactions and contributions of additional genetic elements, it is possible that aggravating genetic modifiers of CTLA-4 may explain the incomplete penetrance and variable expressivity. To date, there has been one report of functional epistasis where a rare variant in *JAK3* (*10*) appeared to enable expressivity of a *CTLA4* variant (*10*). This *JAK3* variant was not found in a cohort of 52 unrelated CTLA-4h patients (*10*), suggesting that there are likely to be additional modifier genes.

Here, we report functional epistasis between DECTIN-1, a pattern recognition receptor (PRR) specific to fungal components, and CTLA-4 in a severe case of CTLA-4h. Whole genome sequencing (WGS) revealed a maternally inherited pathogenic *CTLA4* variant together with a paternally inherited rare (MAF = 0.004) heterozygous variant in the gene encoding DECTIN-1, *CLEC7A* (L183F). Beyond its role in fungal immune surveillance, DECTIN-1 is also known to regulate gut microbial composition and functionality. Indeed, DECTIN-1–deficient mice (*Clec7a^−/−^*) exhibit heightened susceptibility to chemically induced colitis, while human polymorphisms in the DECTIN-1 gene (*CLEC7A*) are strongly associated with ulcerative colitis (*11*).

We report that DECTIN-1 L183F inhibited protein dimer formation, localization, and ligand binding. Importantly, DECTIN-1 stimulation increased peripheral T_reg_ differentiation of human and mouse T cells in vitro and in vivo, suggesting that partial loss of DECTIN-1 function may explain the full IDAIL expressivity in the patient (*12*). Our data reveal a previously unappreciated epistatic interaction between CTLA-4 and DECTIN-1 that maintains immune homeostasis by respective control of T_reg_ quality and quantity.

## RESULTS

### Identification of the *CTLA4* and *CLEC7A*/DECTIN-1 variants

We conducted WGS on a 20-year-old Spanish proband (only child), who exhibited classical symptoms of IDAIL, including early-onset type 1 diabetes (diagnosed at 15 months old), severe enteritis, genital vitiligo and atopic dermatitis. Throughout his childhood, he faced recurrent respiratory infections, including pneumonia, alongside pronounced reactive hypereosinophilia, which constituted up to approximately 65% of total peripheral blood mononuclear cells (PBMCs) at times. Notably, at the age 13, he experienced severe diarrhea and ascites, accompanied by eosinophil infiltration in the esophagus, stomach, and bone marrow. Medical investigations revealed a clonal γδ T cell band, characterized as reactive, with subsequent exclusion of FIP1L1-PDGFRA and PDGFRB rearrangements, as well as any abnormal karyotype. Over time, he developed esophageal candidiasis and sepsis due to *Salmonella typhi* and *Clostridium difficile* infection, which was accompanied by a gradual development of hypogammaglobulinemia. A complete clinical case description is included in the Supplementary Materials.

**Fig. 1.**
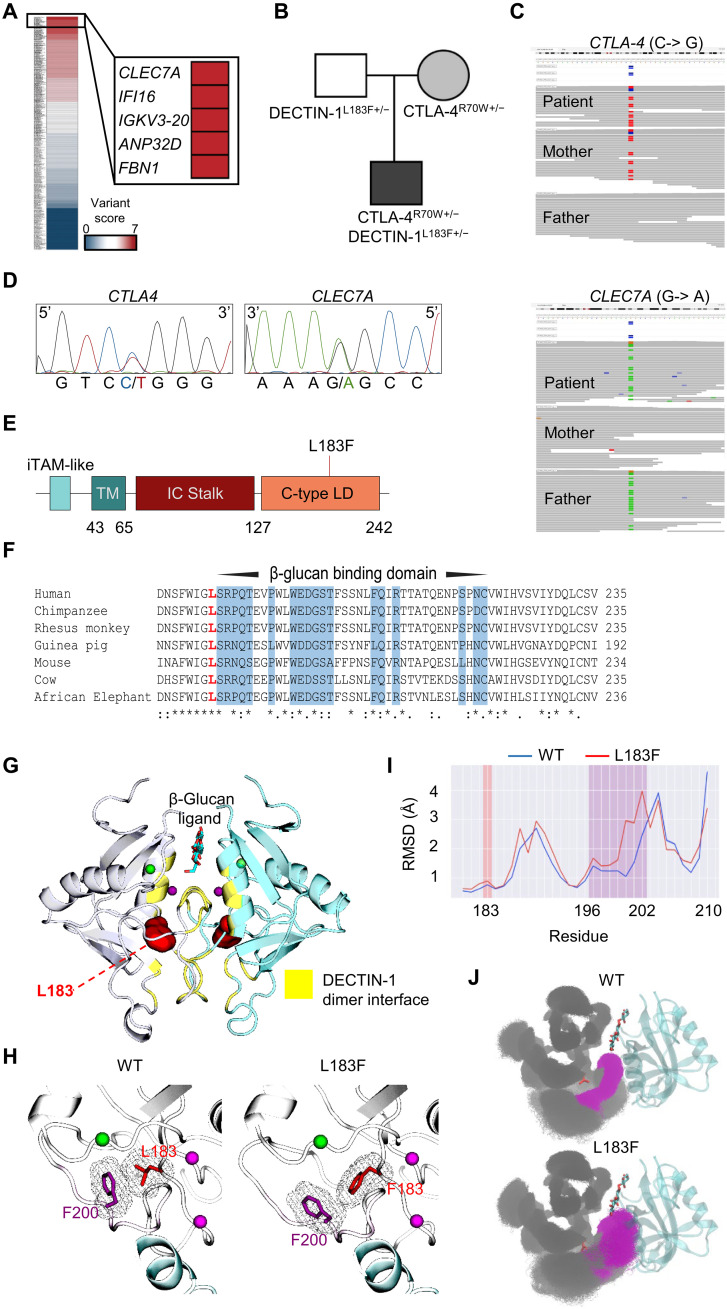
Identification of CTLA4 and *CLEC7A*/DECTIN-1 variants. (**A**) Heatmap showing variant scores calculated by a variant prioritization algorithm from WGS in the proband. (**B**) Family pedigree depicting the maternally inherited CTLA-4 and paternally inherited DECTIN-1 variants. (**C**) IGV viewer snapshots showing WGS read depth of the kindred in *CTLA4* and *CLEC7A* regions. The heterozygous paternally inherited *CLEC7A* variant is shown in green while the maternally inherited *CTLA4* variant is in blue. (**D**) Chromatograms showing the *CTLA4* (C>G: forward strand, Chr 2) and *CLEC7A* (G>A: reverse strand, Chr 12) variants in the patient through Sanger sequencing. (**E**) Protein domain schematic of DECTIN-1 with the L183F mutation (red line). (**F**) Sequence alignment analysis showing conservation of DECTIN-1. Residues associated with the dimer interface responsible for β-glucan binding are highlighted in blue while the variant position (L183F) is bolded in red. (**G**) A visual representation of murine DECTIN-1 dimer crystal structure [PDB ID: 2cl8 (*19*)] (silver and cyan) with bound β-glucan (licorice), between the dimer interface (yellow). L183 is depicted in its hydrophobic pocket (red). (**H**) Interaction between L/F183 (red) and nearby F200 (purple) residues, showing the displaced F200 residue and loop in the mutant state. (**I**) RMSD plot for human DECTIN-1, with L/F183 (red) and the F200-containing loop (purple) highlighted. (**J**) WT and L183F monomer snapshots (gray) superimposed against a reference binding partner (cyan), with attention drawn to the F200 loop region (purple, residues 196 to 202). Structural images displayed are generated with VMD and PyMOL software.

Bioinformatic analysis revealed a known pathogenic maternally inherited missense variant in *CTLA4*, c.208C>T p.R70W, confirmed by Sanger sequencing (Fig. 1, A to D). This heterozygous variant has been previously reported to be causative of CTLA4-h with incomplete penetrance (*1*, *2*). The R70W variant was also present in the patient’s mother who had been diagnosed with mild sarcoidosis, dysphagia with eosinophilic infiltrates of esophagus, low IgM, and decreased percentages of memory B cells.

The severity of the patient’s enteritis and additional clinical manifestations (e.g. chronic salmonellosis and quite remarkable severe persistent hypereosinophilia) prompted us to search for additional disease-contributing or modifying variants (*1*, *2*). We first ruled out the possibility of a second somatic variant in *CTLA4* through high-coverage whole genome sequencing of sorted peripheral T cells. We then ranked all WGS-identified variants through an established prioritization algorithm that considers allele frequency, in silico pathogenicity predictions, and reported functions in the immune system (*13*). These analyses revealed a rare (MAF = 0.004) paternally inherited heterozygous missense variant in *CLEC7A,* c.547C>T (G>A, reverse strand Chromosome 12), leading to a leucine-to-phenylalanine substitution at position 183 (p.L183F) of the DECTIN-1 protein (Fig. 1, A to C).

DECTIN-1 is a well-described PRR with crucial functions in anti-fungal immunity through the recognition of β-glucans, e.g., zymosan, a naturally occurring component of the cell wall of *Saccharomyces cerevisiae* (*14*). DECTIN-1 can also recognize bacterial antigens including *Salmonella* (*15*, *16*). In contrast with this role in anti-microbial immunity, DECTIN-1 recognition of commensal species can induce tolerogenic immune responses by promoting differentiation of regulatory antigen-presenting cells (DCs and macrophages) that help maintain gut symbiosis (*14*, *17*, *18*). This has been thought to underpin the observed association of increased severity of ulcerative colitis with DECTIN-1 polymorphisms (*11*).

Given the proband’s history of infections and/or antibody responses to microbes known to bear DECTIN-1 ligands (*Saccharomyces*, *Candida*, *Salmonella*, and *Clostridium*) and unusually severe enteritis, the *CLEC7A L183F* allele emerged as a promising candidate to increase expressivity of the *CTLA4* allele and/or modify the IDAIL phenotype. Sanger sequencing confirmed the presence of *CLEC7A* L183F in the patient (Fig. 1D), and in silico tools predicted that this variant was highly deleterious (Polyphen 2 = 1, CADD = 25.5, Sift = 0).

### DECTIN-1 L183F prevents dimerization of the DECTIN-1 receptor

DECTIN-1 is only expressed in mammals and is absent from other vertebrates and lower species. Alignment of DECTIN-1 protein sequence from several mammals revealed that lysine-183 is highly conserved (Fig. 1F). L183 is found within the C-type lectin domain and adjacent to the dimer interface that traps the ligand β-glucan (*19*). Dimer formation is essential to form a functional DECTIN-1, i.e., capable of binding its ligand. Given the location of the L183F substitution, we determined whether it could alter DECTIN-1 dimer formation and, thus, its function. To test this, we conducted molecular dynamics (MD) simulations on murine DECTIN-1 [Protein Data Bank (PDB) ID: 2cl8] and reconstructed human dimeric and monomeric structures in the absence or presence of the L183F substitution (see Supplementary Methods for details) (fig. S1A) (*20*–*23*). Our simulations revealed that L183 is located within a tightly packed hydrophobic pocket identified in the DECTIN-1 crystal structure (Fig. 1G). Introduction of the L183F substitution caused pronounced destabilization of dimer structure, supported by our calculations showing elevated free energy values of mutating L183 to F183, observed for both human and mouse systems (fig. S1B). We specifically observed changes to the packing of side chains in the hydrophobic pocket, wherein L183F pushed against F200 and destabilized the dimer-forming loop region of which F200 is part (Fig. 1H). Destabilization of the loop was confirmed through simulations of DECTIN-1 monomer simulations, wherein the dramatically destabilized F200 loop often projected into space normally occupied by the dimer-partner-protomer in the healthy (L183) state (Fig. 1J), generating pronounced shifts in the position of the loop indicated by its greater root mean square deviation (RMSD) from its dimer forming location (Fig. 1I and fig. S1C). In summary, the L183F substitution may prevent dimer formation by destabilizing the dimer-forming loop region.

### L183F is a LoF mutation in DECTIN-1

**Fig. 2.**
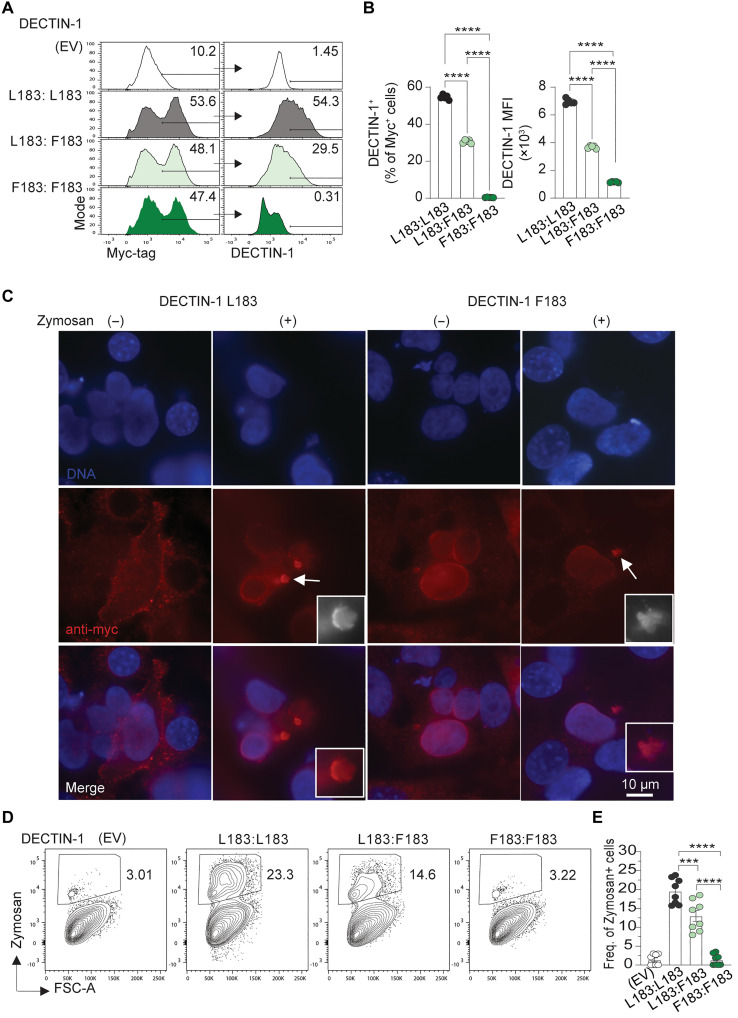
Functional effects of the L183F mutation on DECTIN-1. (**A**) Representative histograms of intracellular DECTIN-1 expression within Myc-tag^+^ (“transfected”) HEK293 cells, transfected with WT (L183:L183), mutant (F183:F183), and a combination of WT and mutant *CLEC7A* DNA (L183:F183). (**B**) Quantification of the frequency of DECTIN-1^+^ cells and DECTIN-1 MFI within Myc-flag^+^ cells (*n* = 5, each dot is a separate transfection). (**C**) Representative immunofluorescence images depicting localization of DECTIN-1 protein in NIH3T3 cells transfected with WT or mutant *CLEC7A*, in the absence or presence of depleted zymosan (100 μg/ml). (**D**) Flow cytometric plots and (**E**) quantification of zymosan uptake through DECTIN-1–mediated phagocytosis in HEK293 cells transfected as in (A) and incubated with pHrodo zymosan particles for 2 hours. Data are pooled from two independent experiments (*n* = 8, each dot is a separate transfection). Statistical significance in (B) and (E) was calculated by one-way analysis of variance (ANOVA), ***P < 0.001 and ****P < 0.0001.

Having predicted that DECTIN-1 L183F prevents dimer formation, we next investigated this experimentally. For this, we used an anti-DECTIN-1 antibody clone (REA515) known to only bind the 28-kDa dimer, and not the monomer (*19*). HEK293 cells transfected with a Myc-tagged mutated DECTIN-1 exhibited a marked loss of DECTIN-1 dimer expression both on the cell surface and in intracellular compartments (Fig. 2, A and B, and fig. S1, D and E). Cells transfected with equal amounts of wild-type (L183) and mutant protein (F183) to mimic the heterozygous state expressed approximately half the amount of dimer DECTIN-1 compared to cells transfected with WT:WT (L183:L183) protein (Fig. 2, A and B, fig. S1, D and E). Barely any cells transfected with only mutant protein (F183:F183) expressed DECTIN-1, suggesting that as predicted, there was impaired dimer formation. Expression of the Myc-tag reporting the presence of total (monomeric or dimeric) WT or mutant DECTIN-1 protein was largely comparable (Fig. 2A), suggesting that only the dimer DECTIN-1 was compromised.

To determine whether L183F alters DECTIN-1 localization we transfected the Myc-tagged DECTIN-1 construct into NIH3T3 cells and stained for Myc to allow detection of total DECTIN-1. At steady state, the WT DECTIN-1^L183^ protein could be detected within the cytoplasm and on the cell surface of expressing cells. DECTIN-1^F183^ failed to localize to the cell surface membrane, and was largely perinuclear, presumably within the endoplasmic reticulum, suggesting that lack of dimer formation also perturbs protein trafficking to the cell membrane (Fig. 2C).

We also stimulated these cells with depleted zymosan, which, when treated with NaOH, abrogates its interaction with TLR-2 (*14*). Zymosan stimulation resulted in WT DECTIN-1 assembling into projected clusters, which were well defined and extended from the membrane, indicative of phagocytosis (Fig. 2C). In contrast, this phenomenon was severely impaired in cells transfected with DECTIN-1^F183^, again indicative of a LoF mutation.

To investigate DECTIN-1 function, we next conducted phagocytosis assays on transfected HEK293 cells and incubated them with pH-sensitive–conjugated zymosan particles that fluoresce only when internalized through phagocytosis. DECTIN-1^F183^ was unable to phagocytose zymosan particles, and like our previous findings, this effect was sensitive to gene dose (Fig. 2, D and E). Collectively, these findings suggest that DECTIN-1 L183F is a LoF variant, compromising both DECTIN-1 dimerization, expression, membrane localization and phagocytosis.

### DECTIN-1 deficiency is associated with decreased T_reg_ frequency

**Fig. 3.**
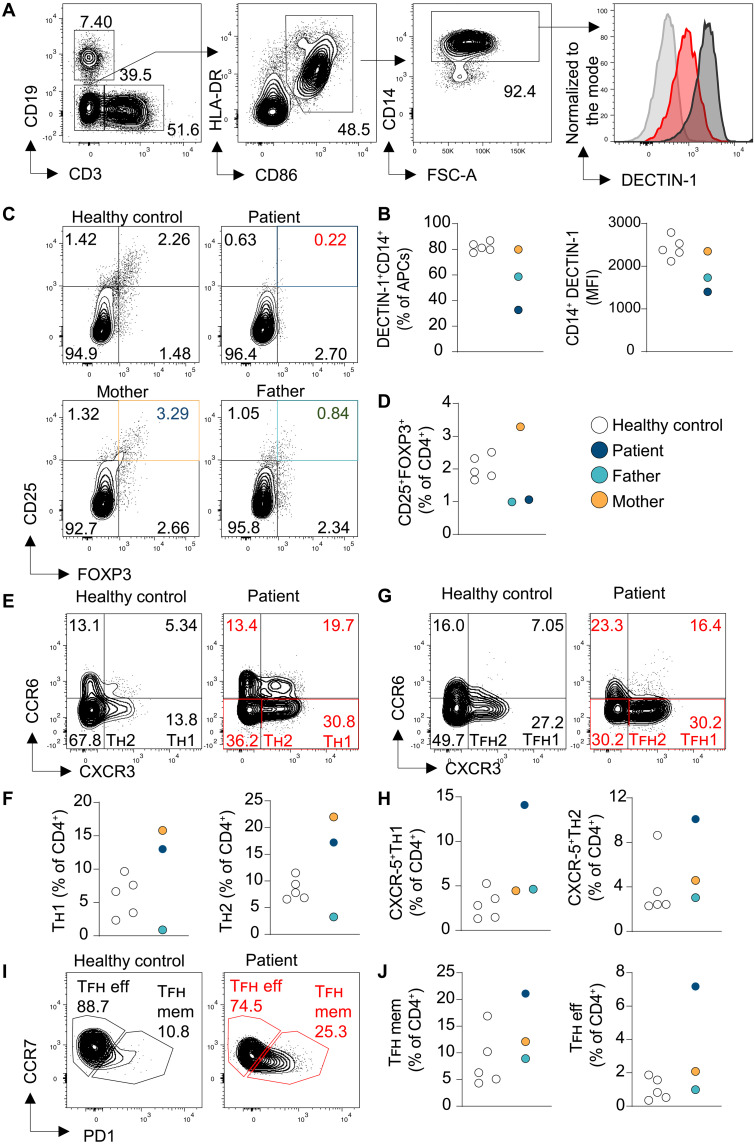
DECTIN-1 expression and immunophenotyping of the kindred. (**A**) Flow cytometric plots showing gating strategy for detecting DECTIN-1 expression on monocytes (CD14^+^) from PBMCs. (**B**) Quantification of the frequency of DECTIN-1^+^ cells among CD14^+^ monocytes and DECTIN-1 MFI in CD14^+^ monocytes from PBMCs in the proband (red), a healthy control (dark gray), and an FMO (light gray). (**C**) Flow cytometric plots, and (**D**) quantification of T_regs_ (CD25^+^FOXP3^+^) from the patient and healthy controls. (**E** to **H**) Representative flow cytometric profiles and quantified frequencies of CXCR5^−^/CXCR5^+^ T_H_1 (CXCR3^+^CCR6^−^) and T_H_2 (CXCR3^−^CCR6^−^) CD4^+^ cells and CXCR5^+^ (T_FH_) effector (CCR7^+^) and memory (PD1^+^) cells from PBMCs of the kindred and healthy blood donors. Each dot is representative of a separate PBMC donor. In (**F** to **J**), the colors are the same as those in the legend for (B) and (D).

Having established that the patient has inherited two LoF variants in *CTLA4* and *CLEC7A*, we next interrogated whether these variants affected protein expression in the patient. Immunophenotyping of PBMCs isolated from the kindred and healthy controls revealed that T_reg_ CTLA-4 expression was slightly decreased in the patient and his mother, both of whom carry the *CTLA4* R70W variant (fig. S2, A to C). Previous studies have reported that this variant predominantly prevents receptor binding to CD86 (*1*). We also assessed DECTIN-1 expression and given that monocytes exhibit the highest amount of DECTIN-1 expression among PBMCs, they were used as a positive control of DECTIN-1 expression. DECTIN-1–expressing cells as a frequency of CD14^+^ monocytes, or DECTIN-1 mean fluorescence intensity (MFI) per monocyte, were decreased in the patient and his father, who both bear the L183F mutation (Fig. 3, A and B). In contrast, the patient’s mother and healthy controls expressed DECTIN-1 comparable to that in healthy controls. These results are consistent with our demonstration of defective surface expression of DECTIN-1 L183F conferred by the functional defect in dimerization.

We next sought to characterize the immune cell subsets involved in the pathogenesis of IDAIL in the patient. The first notable variation was a decrease in FOXP3^+^ cell frequencies in both the patient and his father (Fig. 3, C and D). Damaging *CTLA4* variants typically result in a compensatory increase in T_reg_ numbers in mice, and has been reported in some humans (*24*). As seen in other patients carrying the R70W *CTLA4* variant, the patient’s mother had increased T_regs_ (Fig. 3, C and D). Additional immunophenotyping on the kindred and healthy controls revealed an expansion of T_H_1 and T_H_2 effector populations in both the patient and his mother (Fig. 3, E and F), concordant with previous descriptions of patients with CTLA-4h (*1*, *9*). However, unlike his mother, the patient exhibited an increase in CXCR5^+^ T_H_1 and T_H_2 effector cells, which have been associated with interferon-γ (IFN-γ)–mediated IDAIL and increased B cell help (Fig. 3, G and H) (*25*). This was accompanied by elevated T follicular helper (T_FH_) cell frequencies (Fig. 3, I and J). Given that both the patient and the father harbor the L183F DECTIN-1 mutation, we considered the possibility that DECTIN-1 might be important in sustaining T_reg_ cell numbers, which, if defective, may act in epistasis with CTLA-4 deficiency, and cause the observed increase in effector T cells in the patient.

### DECTIN-1 facilitates conventional and γδ T_reg_ cell differentiation

**Fig. 4.**
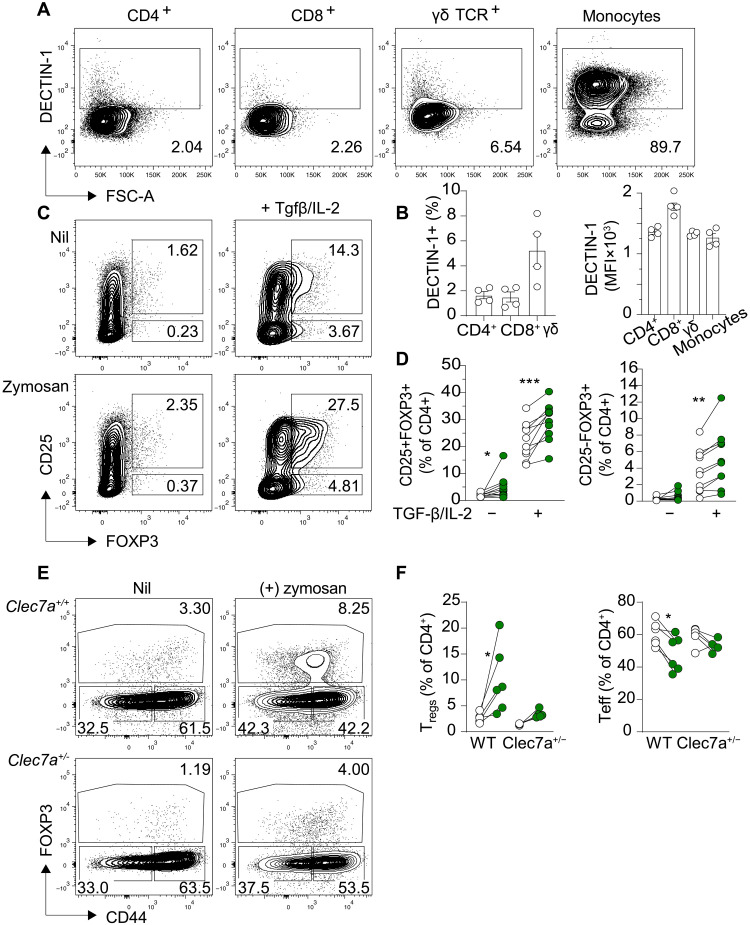
DECTIN-1 induces T_reg_ differentiation. (**A**) Flow cytometric plots and (**B**) quantification of DECTIN-1 expression in monocytes and the indicated T cell subsets following α-CD3/α-CD28 stimulation. (**C**) Flow cytometric plots and (**D**) quantification of induced T_regs_ (CD25^+^FOXP3^+^CD4^+^), from MACS-sorted human naïve T cells cultured with rTGF-β (5 ng/ml) and rIL-2 (1 mg/ml) in the presence or absence of zymosan (100 μg/ml) for 7 days. Each dot represents the mean value of cultures set up in triplicate from a single healthy blood donor. (**E**) Flow cytometric plots and (**F**) quantification of the proportion of murine iT_regs_ induced from FACS-sorted WT or Dectin-1 haploinsufficient (*Clec7a^+/−^*) naïve T cells, following plate bound α-CD3 (3 μg/ml), soluble α-CD28 (2 μg/ml), and rIL-2 (5 ng/ml) stimulation, activation in the absence or presence of zymosan (100 μg/ml) for 3 days. Data are representative of two independent experiments (*n* = 6). Statistical significance in (D) and (F) was calculated by nonparametric paired *t* tests, *P < 0.05, **P < 0.01, and ***P < 0.001.

Having observed an association between decreased T_regs_ and the L183F DECTIN-1 mutation, we considered the possibility that DECTIN-1 could influence T_reg_ cell differentiation. We first assessed DECTIN-1 expression on activated CD4^+^, CD8^+^, and γδ TCR^+^ T cells following anti-CD3 and anti-CD28 activation of magnetically sorted CD3^+^ cells from healthy blood donors. Among all T cell subsets, DECTIN-1 expression was highest in activated γδ T cells, and while DECTIN-1–expressing CD4^+^ T cells were less frequent, they expressed comparable DECTIN-1 amounts to monocytes (Fig. 4, A and B, and fig. S3A).

Next, we determined whether DECTIN-1 stimulation could influence T_reg_ cell differentiation or function. We conducted a classical T_reg_ differentiation assay by culturing naïve CD4^+^ T cells from healthy blood donors with depleted zymosan as a DECTIN-1 stimulus. DECTIN-1 signaling increased formation of FOXP3^+^ T_regs_ (both CD25^+^ and CD25^−^ cells) from naive CD4^+^ T cells (Fig. 4, C and D). Zymosan-induced T_reg_ (zymosan iT_reg_) cell expansion was observed in the presence or absence of T_reg_-inducing cytokines, transforming growth factor–β (TGF-β) and interleukin-2 (IL-2), in a dose-dependent manner (fig. S3B). We validated the suppressor ability of these induced T_regs_ in conventional T_reg_ suppression assays: zymosan-induced T_regs_ were as suppressive as conventionally induced (control) T_regs_ (fig. S3C). DECTIN-1 also boosted mouse T_reg_ cell differentiation (Fig. 4, E and F, and fig. S3D) independently of TGF-β signaling, thus uncovering an evolutionarily conserved pathway of T_reg_ cell induction.

### DECTIN-1 signaling acts in γδ T cells to limit IL-5 release

**Fig. 5.**
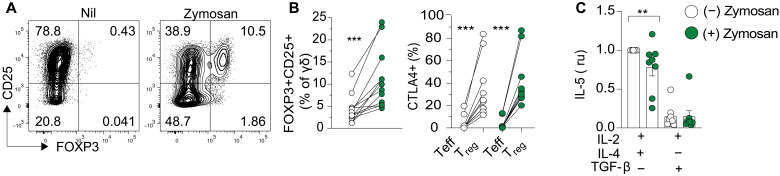
DECTIN-1 signaling acts on γδ T cells to limit IL-5 release. (**A**) Flow cytometric plots and (**B**) quantification of induced γδ T_regs_ (FOXP3^+^CD25^+^) from MACS-sorted naïve γδ T cells activated with α-CD3/α-CD28 beads (1:1) together with rIL-2 with or without zymosan (100 μg/ml) for 10 days. (**C**) Quantification of IL-5 in culture supernatants of γδ T cells activated in T_H_2 [rIL-2/rIL-4 (50 ng/ml)]– or T_reg_ [rIL-2/rIL-15(50 ng/ml)/rTGF-β]–inducing conditions. MFI values are normalized to the T_H_2-inducing conditions. Human in vitro T cell differentiation data are representative of three independent experiments (*n* > 8), where each dot represents the mean value of cultures set up in triplicate from a single healthy blood donor. Statistical significance in (B) and (C) was calculated by paired *t* tests, **P < 0.01 and ***P < 0.001.

Given that the patient originally presented with a reactive clonal γδ T cell population, we next interrogated whether DECTIN-1 could also influence γδ T cells. Similar to our previous observations, zymosan stimulation increased the number of CD25^−^ γδ T_regs_ derived from naïve γδ T cells from healthy blood donors (fig. S3E). Surprisingly, zymosan stimulation alone was also able to induce naïve γδ T cells to differentiate into FOXP3^+^ γδ T_regs_ in the absence of TGF-β (Fig. 5, A and B). Additionally, these γδ T_regs_ expressed more CTLA-4 compared to their naïve progenitors, concordant to previous findings describing up-regulation of regulatory markers (CTLA-4 and CD69) in γδ T_regs_ (Fig. 5B) (*26*). Together, these results suggest a role for DECTIN-1 in promoting FOXP3 expression and T_reg_ cell differentiation.

A puzzling phenotype in the patient was the severe reactive hypereosinophilia—uncharacteristic of IDAIL due to CTLA-4h, with abundant eosinophilic infiltrates even after treatment for tuberculosis infection, known to induce an eosinophil response (*27*, *28*). Hypereosinophilia typically occurs in the presence of elevated IL-5. In humans, γδ T cells play important roles in eliciting the eosinophil influx that occurs during allergic responses and appear to express vast amounts of *IL5* mRNA (BIOGPS, primary cell atlas, 207952_at) (*29*). The patient exhibited a γδ T cell band shown to be reactive, suggesting the possibility that activated IL-5–producing γδ T cells may contribute to the hypereosinophilia (*29*–*31*). IL-5 was not increased in the proband’s serum in a single measurement while on steroid treatment and limited access to PBMCs from the patient precluded enumeration of IL-5–producing γδ T cells. We nevertheless investigated whether DECTIN-1 may modulate IL-5 production by γδ T cells. Stimulation of human γδ T cells with IL-4 plus IL-2 significantly promoted IL-5 production whereas this was inhibited in the presence of the DECTIN-1 ligand zymosan (Fig. 5C). Furthermore, γδ T_regs_ induced in the presence of the DECTIN-1 ligand did not produce IL-5 (Fig. 5C). These results suggest that DECTIN-1 activation may control IL-5 production by both increasing γδ T_regs_ (that do not produce IL-5) and directly limiting IL-5 release from effector γδ T cells. It is therefore possible that DECTIN-1 haploinsufficiency in the patient may contribute to hypereosinophilia.

### Decreased T_reg_ differentiation and function in combined DECTIN-1/CTLA-4 hemizygosity

**Fig. 6.**
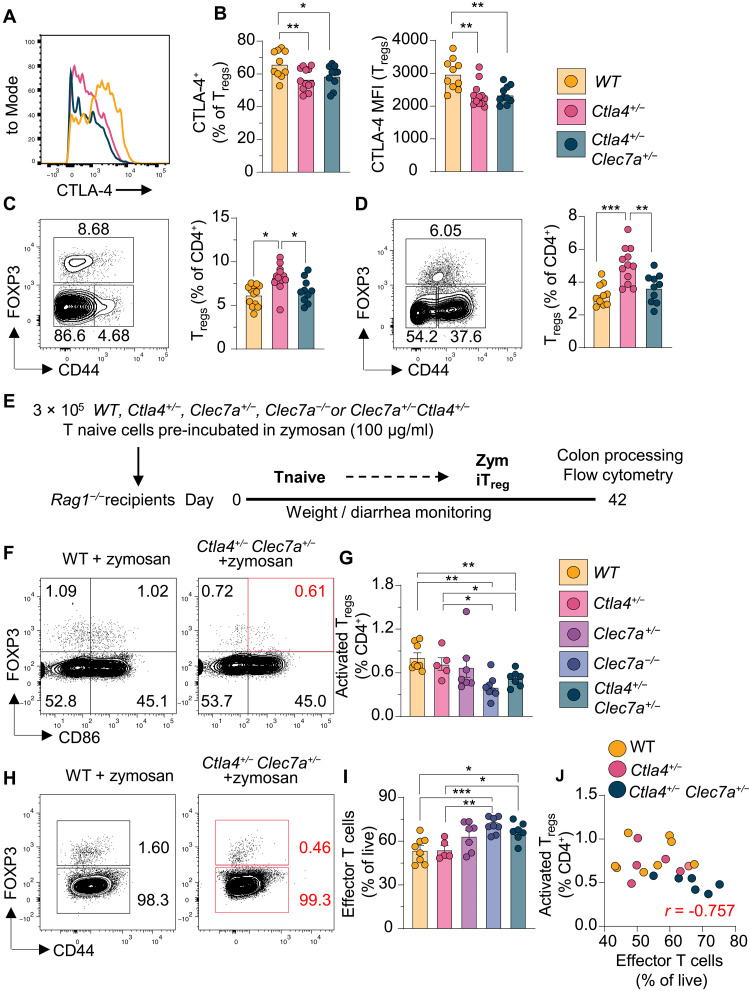
Decreased T_reg_ differentiation and function in combined DECTIN-1/CTLA-4 deficiency. (**A**) Flow cytometric histograms and (**B**) quantification of CTLA-4 expression in splenic CD4^+^ T_regs_ from WT, *Ctla4^+/−^*, and *Ctla4^+/-^Clec7a^+/−^* mice. (**C** and **D**) Flow cytometric plots and quantification showing frequencies of peripheral T_reg_ (FOXP3^+^CD44^+^) from mice before (C) and 7 days after (D) zymosan (100 μg/ml, i.p.) injections. Bars represent medians and each dot denotes a single mouse. Data are representative of two independent experiments (*n* = 10). (**E**) Schematic of the *Rag1^−/−^* adoptive transfer model of zymosan-mediated T_reg_ expansion. (**F** to **I**) Representative flow cytometric plots and quantified frequencies of activated T_reg_ cells (CD86^+^FOXP3^+^) (F and G) and T effector (FOXP3^−^CD44^+^) (H and I) in *Rag1^−/−^* recipient mice (with zymosan) as in (E). (**J**) Pearson correlation analyses between T effector and activated T_regs_ in WT (*r* = 0.4), *Ctla4^+/−^* (*r* = 0.3), and *Ctla4^+/−^Clec7a^+/−^* (*r* = −0.757) recipients (*n* = 5 to 10 per genotype). Statistical significance in (B) to (D) and (G) to (I) was calculated with nonparametric Mann-Whitney tests, *P < 0.05, **P < 0.01 and ***P < 0.001.

Having uncovered an immunomodulatory role of DECTIN-1 in promoting T_reg_ cell differentiation, we next determined whether DECTIN-1 may modify the phenotype conferred by *Ctla4* heterozygosity. For this, we generated mice heterozygous for LoF alleles in both *Clec7a* (Dectin-1) and *Ctla4* (*Clec7a^+/-^Ctla4^+/−^*) (Fig. 6, A and B, and fig. S4). As described in human CTLA-4h and the proband’s mother carrying the *CTLA4* R70W variant, *Ctla4^+/−^* mice displayed a compensatory increase in peripheral blood T_regs_ compared to WT mice (*1*, *2*, *9*, *24*) (Fig. 6C). As observed in the proband, *Clec7a* deficiency prevented the corresponding T_reg_ increase in *Ctla4^+/-^Clec7a^+/−^* mice (Fig. 6C). This phenomenon was also observed and magnified after 7 days of intraperitoneal zymosan administration: *Ctla4^+/−^* mice had a ~2-fold increase in splenic T_regs_ compared to *Clec7a^+/-^Ctla4^+/−^* mice (Fig. 6D and fig. S5A), again suggestive of a role for DECTIN-1 in promoting T_reg_ formation.

While our previous in vitro experiments have shown a clear role for DECTIN-1 in inducing T_reg_ formation from naïve T cells, we sought to investigate whether DECTIN-1 signaling in T cells translated into a change in T_regs_ or T effector numbers in vivo. For this, we incubated FACS (fluorescence-activated cell sorting)–sorted naïve T cells from WT, *Ctla4^+/−^*, *Clec7a^+/−^, Clec7a^−/−^* or *Clec7a^+/-^Ctla4^+/−^* mice with depleted zymosan for 30 min to activate Dectin-1, and adoptively transferred them into *Rag1*^−/−^ mice (Fig. 6E). Six weeks later, we enumerated T_regs_ and effector T cells within the colon, where lymphopenia-induced T cell activation occurs (*32*). Although no significant changes in body weight, colon length, or total T_reg_ numbers were observed between *Ctla4^+/−^* and *Clec7a^+/-^Ctla4^+/−^* mice (fig. S5, B to L), there was a significant decrease in recently activated CD86^+^ T_regs_ derived from *Clec7a^+/-^Ctla4^+/−^* T cells, compared to recipients of *Ctla4^+/−^* T cells (Fig. 6, F and G). This decrease in activated T_regs_ correlated with a concomitant increase in T effector/memory cells (FOXP3^−^CD44^+^) in recipients of *Clec7a^+/-^Ctla4^+/−^* cells (*r* = −0.757), compared to recipients of *Ctla4^+/−^* cells (*r* = 0.3) (Fig. 6, H to J, and fig. S5H). Note that, in this model, all transferred naïve T cells become effectors, with high expression of CD44. Together, these results support a role for DECTIN-1 signaling in T_reg_ control of effector T cells in CTLA-4h.

## DISCUSSION

Our work unveils functional epistatic interactions between *CLEC7A* (encoding DECTIN-1) and *CTLA4*, shedding light on their roles in maintaining immune homeostasis and revealing DECTIN-1’s unanticipated contribution to peripherally induced T_reg_ differentiation. Our data suggest that partial loss of DECTIN-1 in a patient with CTLA-4h may enhance IDAIL penetrance and confer additional unique phenotypes, with persistent marked hypereosinophilia as the most remarkable uncommon clinical manifestation.

The nondimerizing nature of the DECTIN-1 L183F variant and its compromised role in microbial phagocytosis may explain the difficulty to clear infections with bacteria coated with DECTIN-1 ligands, and potentially contributes to the proband’s *Salmonella* and *Clostridium* infections and elevated anti-*Saccharomyces* (*ASCA*) antibody titers in the absence of other signs of inflammatory bowel disease. Although partial loss of DECTIN-1 alone may not suffice to elicit these clinical phenotypes, CTLA-4h and the associated immunodeficiency may bring about their expressivity as well as the typical IDAIL phenotypes. For instance, elevated CXCR5^−^ T_H_1 and T_H_2 cells, also seen in the mother, have been observed in both affected individuals and carriers of CTLA-4h variants (including R70W) (*1*). In contrast, expansion of CXCR5^+^ T_H_1 and T_H_2 cells in the patient alone was more interesting, as these populations are associated with autoimmune pathology (e.g., IFN-γ–mediated IDAIL) and chronic infection. One might speculate that expansion of circulating CXCR5^+^ T_H_1 cells, which produce high amounts of IFN-γ, might have been triggered by the patient’s chronic salmonellosis or intestinal dysbiosis (*25*, *33*).

Our clinical case demonstrated a dual presentation: conventional CTLA-4h manifestations combined with an unusually severe reactive hypereosinophilic syndrome (L-HES). This syndrome involves dysregulated “T cell” production of IL-4, IL-5, and/or IL-13, with IL-5 as the principal driver of eosinophilic overproduction (*34*–*36*). Notably, we found that DECTIN-1 signaling suppressed γδ T cell–derived IL-5, implying that partial loss of DECTIN-1 in the patient could contribute to hypereosinophilia.

Our work has also revealed intriguing findings regarding T_reg_ dynamics. We observed a reduction in T_regs_ both in the patient and his father, which contrasts with the increased T_reg_ numbers documented in CTLA-4h patients and *Ctla4^+/−^* mice, as well as in the patient’s mother (*1*, *9*, *24*). Notably, this increase in T_regs_ was absent in double *Ctla4^+/−^* and *Clec7a^+/−^* heterozygous mice, suggesting that DECTIN-1 alone may play a compensatory role by promoting T_reg_ differentiation. It follows that combined *CLEC7A*/DECTIN-1 haploinsufficiency in the patient may lead to loss of “T_reg_ compensation,” resulting in increased expressivity of the CTLA-4 variant in this kindred. Moreover, we have shown that DECTIN-1 signaling directly promotes the induction of T_regs_ from both αβ and γδ T cell precursors. The loss of compensatory T_regs_ due to partial DECTIN-1 deficiency might underlie the observed expansion of γδ^+^ and CD4^+^ effector T cells in the patient. This is in line with our experiments demonstrating that inhibition of DECTIN-1–mediated T_reg_ differentiation can exacerbate effector T cell expansion. Our in vivo T cell transfer experiments support the notion that attenuated DECTIN-1 signaling reduces the ratio between activated (CD86^+^) *Ctla4^+/−^* T_regs_ and effector T cells. Equally, attenuation of DECTIN-1 signaling in the patient may increase expressivity of the *CTLA4* variant.

We were intrigued to find that DECTIN-1 can promote T_reg_ formation in vitro even in the absence of TGF-β. This observation is noteworthy considering that other molecules known to induce T_regs_, such as retinoic acid, drive T_reg_ differentiation through mechanisms that are dependent on TGF-β-driven Smad3 signaling (*37*). While this role of DECTIN-1 in mediating T_reg_ function has not been described, it is not entirely unexpected, as DECTIN-1 signaling is known to induce both immunogenic and regulatory signaling pathways. For example, DECTIN-1 signaling drives anti-fungal IL-17 responses through activation of the NF-κB pathway, but can also induce tolerogenic APCs by increasing NFAT expression and secretion of regulatory cytokines such as IL-10 and IL-12p70 (*14*, *38*, *39*). Given that NFAT expression plays a crucial role in the induction of peripheral T_regs_, it is plausible to speculate that NFAT-mediated mechanisms might be involved in DECTIN-1–mediated T_reg_ induction as well.

The restricted expression of DECTIN-1 on a minor fraction of CD4 T cells is intriguing. It is possible that DECTIN-1–expressing CD4 T cells may represent a specialized subset with distinct immunoregulatory properties. Given DECTIN-1’s role as a PRR for fungal components, it is conceivable that this subset could be enriched in tissues prone to encounter fungal antigens, such as mucosal surfaces or barrier tissues. A recent study revealed that γδ T cells within the lamina propria (LP) up-regulated DECTIN-1 expression under conditions of chronic social-defeat stress (*40*). The authors reported dynamic expression patterns of DECTIN-1, which suggests that environmental cues can modulate its expression on γδ T cells. Whether the same is true for DECTIN-1–expressing CD4 T cell subsets remains unknown.

While our study illuminates functional epistasis between *CLEC7A* and *CTLA4* in CTLA-4h, we must recognize certain limitations that guide future directions. Although we have revealed functional interactions between CTLA-4 and DECTIN-1 in T_reg_ biology, we cannot exclude the influence of alternative factors such as additional genetic modifiers and environmental variables on the patient’s CTLA4-h presentation. Also, while insights from combined heterozygosity for *Ctla4* and *Clec7a* LoF alleles in mice have been informative, the absence of IDAIL in these mice and, more generally, the absence of phenotypes in heterozygous *Ctla4*^+/−^ mice—unlike human CTLA-4h—hamper a comprehensive exploration of how DECTIN-1 modifies CTLA4-h in vivo. It is possible that the controlled specific pathogen–free conditions under which they are housed limit the presence of certain gut bacteria required for the penetrance and expressivity of pathogenic CTLA-4 variants.

The identification of genetic modifiers of CTLA-4 holds promising clinical implications, particularly in guiding the selection of checkpoint inhibitor therapies for cancer patients and predicting the likelihood of severe adverse effects. The therapeutic efficacy of CTLA-4-blocking agents, such as ipilimumab and tremelimumab, varies among patients and is often accompanied by the development of autoimmune side effects, including colitis (*41*, *42*). Investigating the presence of additional rare gene variants such as the *CLEC7A* variant in cohorts of patients receiving CTLA-4 antagonists may aid in predicting patient immune responses and potential side effects.

Our work establishes a role for DECTIN-1 in peripheral T cell tolerance. While convergence of CTLA-4 and DECTIN-1 pathways on the induction of functional T_reg_ responses may so far only explain a case of incompletely penetrant CTLA-4h, cross-talk between the two pathways is likely to underpin the tolerogenic effects of certain gut microbial species on T cell responses, which, in turn, may influence the severity of CTLA-4h and the efficacy and side effects of CTLA-4 immunotherapy.

## MATERIALS AND METHODS

### Study design

This study aimed to determine the functional effects of a rare DECTIN-1 mutation in the development of severe autoimmune disease alongside CTLA-4h.

### Human and mouse ethics

Experimental protocols involving human and mouse samples were performed in accordance with the local institution ethics committee, including the Australian National University’s (ANU’s) Animal and Human Experimentation Ethics Committee. Written informed consent by patients and healthy blood donors was obtained through the Centre for Personalized Immunology Program.

### Human blood sample collection and PBMC preparation

Blood donations from healthy blood donors were collected by an authorized clinician at John Curtin School of Medical Research (JCSMR) or the Canberra Hospital, ACT. Peripheral blood samples were drawn into EDTA tubes and PBMCs were isolated by Ficoll-LymphoPrep (STEMCELL Technologies) density gradient centrifugation and frozen in fetal bovine serum (FBS) containing 10% dimethyl sulfoxide (Sigma-Aldrich) by researchers at JCSMR, ANU. Kindred samples (frozen PBMCs) were kindly delivered from Dr. Pilar Martin (Immunology group leader at the National Centre for Cardiovascular Research (CNIC), Madrid, Spain). Male and female healthy donors were aged between 20 and 30 years of old. This age range was applied to minimize any confounding variables and maintain the integrity of our comparisons between the proband and the healthy control group.

### Whole exome sequencing and variant scoring

For whole exome sequencing (WES) analysis, DNA kindred samples were enriched using the Human SureSelect XT2 All Exon V4 Kit and sequenced using the Illumina HiSeq 2000 (Illumina) system. Bioinformatics analysis was performed at JCSMR, ANU. Variants from the proband were scored through a bioinformatics analysis pipeline as described previously (*13*, *19*).

### Mice

Mice were bred and maintained in SPF conditions at the ANU, Canberra, Australia. Estimations of the expected change between experimental and control groups allowed the use of power analysis to estimate the group size that would enable detection of statistically significant differences. Blinding was used for histological analysis. Mice were used from 6 to 26 weeks. *Clec7a^−/−^* mice created by Gordon Brown were ordered from the Jackson Laboratory (B6.129S6-Clec7atm1Gdb/J, strain id: 012337) while the *Ctla4^+/−^* mice were generated in a C57BL/6NCrL background using CRISPR-Cas9–mediated gene editing technology, in accordance with published protocols by Gurumurthy *et al.* (*43*). Briefly, two single guide RNA (sgRNA) (guide 1: 5′-AGAAGTCCTCTTACAACAG GGG-3′ and guide 2: 5′-GTACCCACCGCCATACTTTG TGG-3′) were designed using CCTop and CRISPOR, respectively, in Exons 2 and 4 of the *Ctla4* coding sequence and exhibited the lowest off-targets effect and highest predicted efficiencies. Cas9 protein and guide RNAs (purchased from Integrated DNA Technology-IDT) were delivered to C57BL/6Ncrl fertilized mouse zygotes with a Ribonucleoprotein complex at 50 and 2.5 ng/μl, respectively. The founder mice were genotyped by long-range PCR and Sanger sequencing using the following primer pairs: SF1: 5′-ATCACCAAAGAAGGCGCTGT-3′ and SR1: 5′-TGAAGGACTCCAAACCAAGGA-3′; SF2: 5′-TGAGGTGACAGAGACTGGAAAC-3′ and SR2: 5′-GGAACCACTGGCTATGTCACA-3′. One of the founder mice contained a *Ctla4* allele with an approximately 2 kb (1926 bp) deletion between exon 2 encoding the ligand binding and dimerization domain and exon 4 encoding the cytoplasmic tail of the CTLA-4 protein (fig. S4). The deletion started in the “T” of M134 in exon 2 and extended until C211 of exon 4. Because of the sudden decline in the health of the founder mouse containing the heterozygous *Ctla4* deletion, ovary transplants into C57BL/6NCrL females were performed to generate the *Ctla4^+/−^* strain. Impaired CTLA-4 expression in subsequent generations was detected by genotyping performed by the Australian Phenomics Facility (APF) JCSMR and through flow cytometric analysis on spleen or peripheral blood samples. These knockout strains were used to breed *Clec7a^+/−^*, *Ctla4^+/−^*, and *Ctla4^+/-^Clec7a^+/−^* mice.

### Sanger sequencing

Primers for human *CTLA4* (5′-3′—CGTGGGGATGAAGCTAGAAG, 3′-5′—ATGGCGGTGGGTACATGA) and *CLEC7A* (5′-3′—CCCGGCCTTGCATTCTTTAC, 3′-5′—TCTTAGCTGCTCGACAGAGG) DNA sequencing were used at 10 μM (primer sequences available on request). PCR amplification was carried out using Phusion Hot Start II DNA Polymerase II (Thermo Fisher Scientific). PCR amplicons were electrophoresed and excised bands were purified using the QIAquick Gel Extraction Kit (Qiagen). Sanger sequencing was completed using Big Dye Terminator Cycle sequencing kit v3.1 (Applied Biosystems) using the same primers used for PCR amplification. Sequencing reactions were run on the 3730 DNA Analyze (Applied Biosystems) system at the ACRF Biomolecular Resource Facility, ANU.

### Flow cytometry

Immune cell subsets from single-cell suspensions from thawed PBMCs or mouse organs and tissues were assessed by flow cytometry. The primary antibodies used for human blood included CD3-BUV737 (UCHT1, BD Horizon), CD19-Buv395 (SJ25C1, BD Horizon), CD4-APC (RPA-T4, BioLegend), CD8-AlexaFluor488 (RPA-T8BD Biosciences), γδ TCR-BUV395 (B1-RUO, BD Biosciences), HLA-DR-PERCP (L243, BioLegend), CD86-BV711 (FUN-1, BD Horizon), CD14-BV510 (Mϕp9, BD Biosciences), DECTIN-1-FITC (REA515, Miltenyi Biotec), CD25-BV650 (PC61, BioLegend), FOXP3-PE (259D/C7, BD Biosciences), CTLA-4-BV421 (BNI3, BD Horizon), CCR6-BV510 (G034E3, BioLegend), CXCR-3-PE (G025H7, BioLegend), CXCR-5-APC (RF8B2, BD Pharmigen), CCR7-FITC (150503, BD Biosciences), and PD1-APCY7 (EH12.2H7, BioLegend). Similarly, the primary antibodies for mouse organs and tissues included CD3-APCY7 (17A2, BioLegend), CD4-A700 (RM4-5, BD Pharmigen), CD44-A700 (IM7, BioLegend), CD86-BV605 (GL1, BD Horizon), γδ TCR (UC7-13D5, BioLegend), DECTIN-1-APC (REA154, Miltenyi Biotec), FOXP3-PECY7 (FJK-16s, Thermo Fisher Scientific, Invitrogen) and CTLA-4-BV421 (UC10-4B9, BioLegend). Before staining with surface markers, cells were incubated with mouse (2.4G2, BD Biosciences) or human (Fc1, BD Biosciences) FC block to prevent nonspecific binding, and live-dead fixable dyes (BioLegend, Thermo Fisher Scientific) were used for detecting dead cells. Intracellular antibodies including FOXP3 and CTLA-4 were stained following fixation in line with the eBioscience fixation kit (Thermo Fisher Scientific ref. 00-5523-00). Samples that were acquired before fixation were resuspended in 7-aminoactinomycin D (7AAD) (A1310, Invitrogen). All samples were acquired on the Fortessa or Fortessa X-20 cytometer at the Microscopy and Cytometry Facility, ANU, with FACSDiva (BD, Biosciences) and analyzed with FlowJo v.10 (FlowJo) software.

### Human T_reg_ differentiation and suppression assays

Naïve CD4 T cells were magnetically sorted from frozen PBMC samples (Naïve CD4^+^ T cell isolation kit: 130-094-131, Miltenyi Biotech), and cultured with recombinant TGF-β (5 ng/ml), IL-2 (1 mg/ml), and anti-CD3/anti-CD28 activation beads (T cell activation kit: 130–091-441, Miltenyi Biotech), in the absence or presence of depleted zymosan (100 μg/ml) stimulation. On day 7, cells were collected and assessed for T_reg_ markers (FOXP3, CTLA-4, and CD25) through flow cytometry or counted for T_reg_ suppression assays. Effector CD4^+^ T cells were magnetically sorted from PBMCs of the same donor using the CD4^+^ T cell isolation kit from Miltenyi Biotech (130–096-533) and CD25-Biotin (M-A251, BioLegend) to exclude T_regs_, then stained with a cell trace violet (CTV) proliferation (C34557, Invitrogen). T_regs_ and T effectors were cocultured with CD3/CD28 activation beads at 1:1 to 1:16 ratios, then resuspended in 7AAD to assess CTV expression.

### Human γδ T cell differentiation cultures

Naïve gamma delta T cells were magnetically sorted from frozen healthy blood donor PBMCs using a Miltenyi Biotech γδ isolation kit and activated with anti-CD3/anti-CD28 beads for 12 days and either T_H_2-inducing recombinant IL-4 (50 ng/ml), IL-2 (1 mg/ml), or T_reg_-inducing IL-15(10 ng/ml), TGF-β (5 ng/ml), and IL-2 (1 mg/ml) cytokines, without or without zymosan (100 μg/ml) stimulation. T_reg_ markers, including intracellular FOXP3 and CTLA-4, were detected using flow cytometry of fixed culture cells while IL-5 expression was determined by cytometric bead arrays (CBAs) (Human IL-5 Flex Set: 558278, BD Bioscience) on cell culture supernatant.

### Murine T_reg_ differentiation cultures

Naïve murine T cells were FACS-sorted from splenocyte cell suspensions using CD3, CD4, CD25, and CD62L surface markers. Naïve T cells (2 × 10^5^) were then stimulated with anti-CD3 (plate bound, 3 μg/ml), anti-CD28 (soluble, 2 μg/ml), and recombinant TGF-β (2 ng/ml) and IL-2 (5 ng/ml), in the absence or presence of zymosan (100 μg/ml) for 3 days.

### Cell transfections

Human HEK293 cells (ATCC, CRL-1573) were transfected in a 24-well plate with 0.5 μg WT or mutated (C550T) *CLEC7A* DNA plasmIDAIL (GenScript) using Lipofectamine 2000 reagent (Invitrogen) overnight. Similarly, murine NIH3T3 cells (ATCC, CRL-1658) were seeded in a six-well plate and transfected with 2.5 μg of WT or mutated *CLEC7A* DNA using LTX reagent (Invitrogen). Details for *CLEC7A* plasmIDAIL are listed as follows: WT final contruct-*CLEC7A*_OHu19199c_pcDNA3.1(+)-c-MYC [Clone ID Ohu19199C, accession no. NM_197947.2, Vector; pcDNA3.1(+)-C-Myc, Ampicillin]. Mutant final construct-C550T (encoding L183F)_ pcDNA3.1(+)-c-MYC.

### DECTIN-1 expression and localization in transfected cells

To detect DECTIN-1 dimer expression, transfected HEK293 cells were stained with an anti-DECTIN-1 antibody clone (REA515) known to only bind the 28- to 33-kDa dimer and not monomers (Miltenyi 130-107-690). Surface dimer expression was then assessed through flow cytometry.

For intracellular Myc and DECTIN-1 expression, transfected HEK293 cells were fixed with the eBioscience fixation kit (Thermo Fisher Scientific ref. 00-5523-00), stained overnight with 1:150 dilution of primary mouse anti-myc antibody (9E10, Merck), and then stained with a secondary donkey anti-mouse Alexa 568 antibody (Invitrogen) and anti-DECTIN-1 antibody clone (REA515) the next day for flow cytometric analysis. To assess localization of DECTIN-1, immunofluorescence staining was carried out on transfected NIH3T3 cells that are not known to express endogenous murine DECTIN-1. Following transfection, NIH3T3 cells were stimulated with zymosan (100 μg/ml) for 30 min or left untreated, fixed for 20 min with 3.7% formaldehyde, and stained with 1:150 dilution of primary mouse anti-myc antibody (9E10, Merck) overnight. Cells were washed with phosphate-buffered saline (PBS) + 0.05% Triton X-100, stained with the donkey anti-mouse Alexa 568 antibody (Invitrogen), and mounted using Vectashield with DAPI (4′,6-diamidino-2-phenylindole) (Vector Laboratories). Stained cells were imaged using an Olympus IX 71 inverted fluorescence microscope with DP Controller software (Olympus) and compiled using Adobe Photoshop software.

### Zymosan phagocytosis assay

HEK293 cells transfected with L183F or WT DECTIN-1 DNA were incubated at 37°C for 2 hours with phrodo red Zymosan particles (Thermo Fisher Scientific). Following incubation, the cells were washed with cold PBS, stained for surface DECTIN-1 (REA515, Miltenyi Biotech), and then assessed for zymosan uptake through flow cytometry.

### Zymosan depletion and injections

Depleted zymosan A from *S. cerevisiae* (Sigma-Aldrich) was prepared by boiling in NaOH for 30 min, in line with previous studies (*9*). To trigger zymosan-mediated DECTIN-1 activation, in vivo mice were administered with 200 μl of 100 μg/ml zymosan in PBS through intraperitoneal injection every 24 hours for 7 days. Injected mice were of mixed sexes and 6 to 10 weeks old.

### Generation of murine single-cell suspensions

Single-cell suspensions from murine spleens were generated following mechanical digestion into 70-μm cell strainers and incubation with 1× lysis buffer (10× stock solution; 44.95 g of ammonium chloride-NH_4_Cl, 5 g of potassium bicarbonate-KHCO_3_, and 185 mg of EDTA in 500 ml of MilliQ water). For LP single-cell suspensions, colons were first cleaned to remove fecal matter and rinsed in 1× PBS, and any connective or adipose tissue was removed. Colons were then sliced longitudinally, cut into small (1 to 3 cm) pieces, and shaken vigorously (250 rpm) at 37°C for 30 min in extraction media—30 ml of RPMI, 93 μl of 5% (w/v) dithiothreitol (DTT), 60 μl of 0.5 M EDTA, and 500 μl of FBS per colon. The extraction solution was filtered through 100-μm filters, while remaining tissue was collected and shaken again at 37°C in digestion media (25 ml of RPMI, 12.5 mg of dispase, 50 mg of collagenase D, and 300 μl of FBS per colon) for 30 min. Digested colon segments were then filtered using 70-μm cell strainers to obtain a single cell suspension, which was rinsed and resuspended in RPMI containing 10% FBS.

### T cell transfer enteritis model

Naïve T cells from WT, *Ctla4^+/−^*, *Clec7a^+/−^, Clec7a^−/−^*, or *Clec7a^+/-^Ctla4^+/−^* mice were sorted by FACS from splenic samples using CD3, CD4, CD45RB, and CD25 surface staining. *Rag1^−/−^* mice were intraperitoneally injected with 3 × 10^5^ T naive (CD45RB^+^) cells suspended in zymosan solution (100 μg/ml zymosan in PBS), following a 30-min preinjection incubation at room temperature. Note that control mice were injected with 3 × 10^5^ T naive (CD45RB^+^) and 2 × 10^5^ T regulatory (CD45RB^−^) cells, in the absence of zymosan stimulation (PBS alone). Mixed male and female donor mice were 8 to 12 weeks of age, while recipient *Rag1^−/−^* mice (also mixed sexes), were 6 to 8 weeks of age. All mice were monitored daily, weighed weekly, and culled on day 42 after transfer. Colons were collected for histology or flow cytometric analysis. Blinded scoring of diarrhea incidence after transfer and colon damage in histology samples was performed.

### MD simulations

Please see Supplementary Methods.

### Statistical analysis

Data and statistical analysis was performed using FlowJo 10 and GraphPad Prism 9, respectively. Unless stated otherwise, paired *t* tests (human in vitro assays) or unpaired nonparametric Mann-Whitney *U* tests (mouse experiments) were used to measure significant differences between groups.
