# Supplementary material for: DECTIN-1: A modifier protein in CTLA-4 haploinsufficiency
Source: Sci Adv. 2023 Dec 6;9(49):eadi9566. doi: 10.1126/sciadv.adi9566 (PMC10699772; doi:10.1126/sciadv.adi9566)
Supplement: Supplementary file 1 — Supplementary Text Figs. S1 to S5 Table S1 [file sciadv.adi9566_sm.pdf]

Science Advances
AAAS

Supplementary Materials for

DECTIN-1: A modifier protein in CTLA-4 haploinsufficiency

Cynthia Turnbull *et al.*

Corresponding author: Carola G. Vinuesa, carola.vinuesa@crick.ac.uk; Pablo F. Canete, p.fernandezdecanetenieto@uq.edu.au

Sci. Adv. **9**, eadi9566 (2023)
DOI: 10.1126/sciadv.adi9566

This PDF file includes:

Supplementary Text
Figs. S1 to S5
Table S1

Supplementary Text

Clinical case study

The patient was diagnosed with type I diabetes at 15 months of age. His infancy was largely uneventful, with regular follow-ups and well-controlled glycaemia. However, at 13 years old, he began experiencing episodes of diarrhea, vomiting, and ascites. Blood leukocyte counts revealed a significantly elevated percentage (60.4%) and absolute counts (up to 9.68 cells/ml) of eosinophils, along with moderate hypogammaglobulinemia (IgG 5.52 g/L, IgA 0.46 g/L, IgM 0.36 g/dL), normal IgE and tryptase plasma levels. Further investigation indicated increased leukopoiesis with prominent eosinophilia in bone marrow biopsy, primarily comprising mature forms. Gastrointestinal pan-endoscopy identified eosinophilic infiltrates in the oesophagus and antral gastric mucosa, without involvement in the gastric body, intestinal, or colonic mucosa. This presentation led to a diagnosis of eosinophilic gastroenteritis, with extensive allergen and parasite assessments yielding no concurrent factors. Various non-biological therapies were administered for hyper-eosinophilia, with prednisone effectively managing exacerbations of diarrhea on low doses. Coeliac and thyroid autoimmune conditions were ruled out, and abdominal ultrasounds showed no adenopathies or visceromegaly. Gradually, vitiligo, mild atopic dermatitis, and eczema developed, responding well to combined topical antibiotic and corticosteroid treatment.

At 19 years old, the patient was referred to the Clinical Immunology department for evaluation. An idiopathic hyper-eosinophilic syndrome, compatible with a lymphocytic variant, was diagnosed based on consistent results from a second bone marrow test at 16 years old. The patient's infectious history revealed respiratory infections, bronchospasm, acute sinusitis, and pneumonia. Notably, the patient had proper antibody responses to vaccinations, including SARS-CoV-2, but exhibited suboptimal response to pneumococcal polysaccharide revaccination. Over time, the patient's immunological profile showed progressive decreases in IgG and, notably, complete IgA deficiency. B cell phenotyping remained consistent, with a significant decrease in both unswitched and switched memory B cells and an increase in CD21low B cells. The patient exhibited sporadic peripheral eosinophilia, colonic eosinophilic infiltrates, and occasional episodes of diarrhea despite the absence of clear evidence of intestinal infection or inflammatory bowel disease. Substitutive subcutaneous immunoglobulin treatment was initiated, and considerations for biological therapy with Abatacept +/- anti-IL5 targeting are underway as steroid-sparing options.

Supplementary Materials and Methods

MD simulations

Starting coordinates: Protein structure of mouse DECTIN-1 in complex with a β-glucan (PDB: 2CL8) was sourced from the RCSB PDB as the starting point for all systems. Mouse numbering of protein residues will be used throughout the rest of the system building description. All protein atoms, the crystalised ions and the β-glucan ligand were kept in the structure, while any other coordinates (waters etc.) were deleted. Protonation states for histidine residues were selected based on calculations using PROPKA12,13. Cysteine bridges were determined from the PDB metadata. All other cysteines along with the known cysteine bridges were then checked manually to ensure they seemed likely to be bridged or protonated residues.

Force field parameters: AMBER ff19SB, OPC, and GLYCAM-06 were used for protein, water, and glycan-ligand parameters accordingly (20-23). For ion parameters, the appropriate 12-6-4 OPC set was used from Sengupta et al (22).

System building: System building was executed using tleap in preparation for AMBER22. For monomer systems, one protomer and the glycan-ligand were first deleted from the prepared dimer structure before processing. Systems designated as human maintained the mouse sequence except for two mutations near to the L182 site of interest: V206I and V227I, while mouse systems were left as is. The rest of the residues that were noted as different in human vs. mouse were distant from the site of interest and left in the mouse sequence. The two human mutations were achieved through deletion of the non-shared sidechain atoms and allowing tleap to fill the sidechain with the new designated atoms for the new residues. For TI systems, two overlapping versions of one protomer were processed in tleap simultaneously, with the second including the mutant L182F site. The redundant protein residues were eliminated in parmed processing later. Each system was solvated with a minimum distance of 10 Å from the protein to the system box edge and had 150 mM NaCl added by random replacement of water molecules. The ion count corresponding to this concentration was calculated by two iterations of SLTCAP18 on a randomly selected system: In the first, we input sum protein and glycan-ligand mass as determined by an initial non-solvated tleap run, desired salt concentration, protein net charge, and the corresponding number of water molecules as determined by an initial solvation with tleap. In the second, we updated the number of waters, accounting for the removal of some water from the first pass due to the ion addition. The resulting ion count was used for all systems of a given size, with two variant counts – one for the monomer systems and one for the dimer systems. Finally, systems were post-processed with parmed19 for hydrogen mass repartitioning to facilitate a 4 fs timestep and to appropriately configure the systems for use of the 12-6-4 ion parameters. For systems heading to thermodynamic integration, additional processing parmed by TIMerge removed the redundant overlapping protein atoms. A second set of parmed processed parameters were also generated for TI systems without correct processing of the 12-6-4 ion parameters. These were for strict use in the brief CPU phase of minimisation and pressurisation of the systems, as pmemd.MPI does not support 12-6-4 ion parameters under TI in AMBER22. Later GPU preparatory phases with full 12-6-4 ions allowed correction of any spurious ion interactions caused during these short, restrained CPU runs.

Simulation parameters: Harmonic position restraints with a force constant of 5 kcal/mol/Å2 were applied to CA, C and N backbone atoms in the thermodynamic integration simulations of residues between 117 to 135 and 235 to 244, along with the central heavy sugar ring atoms of the glycan-ligand and the two chloride and two calcium atoms that were shown bound in the crystal structure. The atoms mentioned were not restrained in the monomer systems but remained restrained in TI simulations. Temperature was controlled under a Langevin thermostat with a collision frequency of 2 ps-1 and a target temperature of 310 K. Pressure was maintained using a Monte Carlo barostat with isotropic position scaling and a pressure relaxation time of 0.5 ps, to a target pressure of 1 bar. Systems were run under an AMBER22 compilation of pmemd.MPI for minimization, initial heating and pressurisation, before being switched to pmemd.cuda for all subsequent preparatory and production phases.

System equilibration: During minimisation, heating and pressurisation, all other backbone atoms in the system were harmonically positionally restrained with a force constant of 1 kcal/mol/Å2. All systems were minimised without SHAKE for 10000 steps of steepest descent with CPU code (TI dimer systems) or with a 50/50 mix of steepest decent and subsequent conjugate gradient (monomer systems). A further 1000 steps followed with SHAKE turned on with the same mixture as mentioned prior depending on system type. Systems were then heated and pressurised for 0.1 ns on CPU code to avoid box errors common to pressurising systems running under GPU code. This was completed with a Berendsen barostat and 1 fs timestep to assist in stability. A further 0.025 ns was completed on GPU with a Berendsen barostat, with another 0.025 ns on the production Monte Carlo barostat. All subsequent systems were then to be moved to a 4 fs timestep. The equilibration restraints were then eliminated in linearly reducing restarting increments, from 1 to 0.1 kcal/mol/Å2 over 10 simulations of 0.25 ns length each, and from 0.1 to 0 kcal/mol/Å2 over 10 simulations of 0.25 ns length each. For non-TI systems, 20 replicas were run from minimisation through this sequence for each of four system treatments.

For TI systems, the lambda value used during system equilibration was that corresponding to the wild-type system. The coordinates at the end of equilibration were utilised as the starting point for the neighbouring window, where a 1 ns equilibration run was completed before allowing the next window to use the previous window's equilibration run as its starting coordinate in a series of runs to smoothly prepare all windows across the lambda spectrum for later parallel production runs. Five replicas of each system treatment were run from minimisation through the entire equilibration sequence.

Thermodynamic integration: Relative free energies of the mutations for each protein variant were determined using the method of thermodynamic integration in which the wild-type residue is slowly mutated to the target mutant during a series of simulations. This was done by breaking the process into two legs that describe the energy to mutate the residue in both the monomer and dimer state. The dimer state was mutated under two separate conditions: with the partner protomer as L182, and L182F. This was done in order to estimate what varied energetic effect dimers that are homozygous or heterozygous for the mutation may have, while avoiding running two simultaneous site mutations in the one system which would complicate convergence. Each free energy calculation was calculated in a single step with the non-shared sidechain atoms defined as softcore under AMBER22's second order smoothstep function22. We utilised an alpha value of 0.5 and a beta value of 12 for the softcore parameters and scaled intra-ligand electrostatics with lambda to mitigate spurious gas phase self-interactions.

We further used gti_syn_mass=1, a new input option of AMBER22 described in the AMBER22 manual that facilitates the use of a 4fs timestep despite the presence of vanishing covalent softcore atoms. Five replicates of each leg of the thermodynamic cycle were prepared with their own equilibration phase as detailed earlier. For production runs, data was collected every 1 ps. 41 lambda windows per replica were used to facilitate a smooth integration surface and were evenly distributed from lambda 0 to 1, inclusive of end-points.

Analysis: Thermodynamic integration analysis was conducted using the python packages alchemlyb23 and pymbar24. Data from production regions were subsampled using pymbar's conservative algorithm to ensure data points were decorrelated to acquire accurate error estimates. Data fitting was executed once for each system and leg of the thermodynamic cycle by pooling decorrelated data for each replica by window. The fit was executed using alchemlyb. The final reported value included a summation of both simulation legs, further multiplied by two to estimate the combined effect of two-site mutations, reported with the accumulative standard error.

Fig. S1.

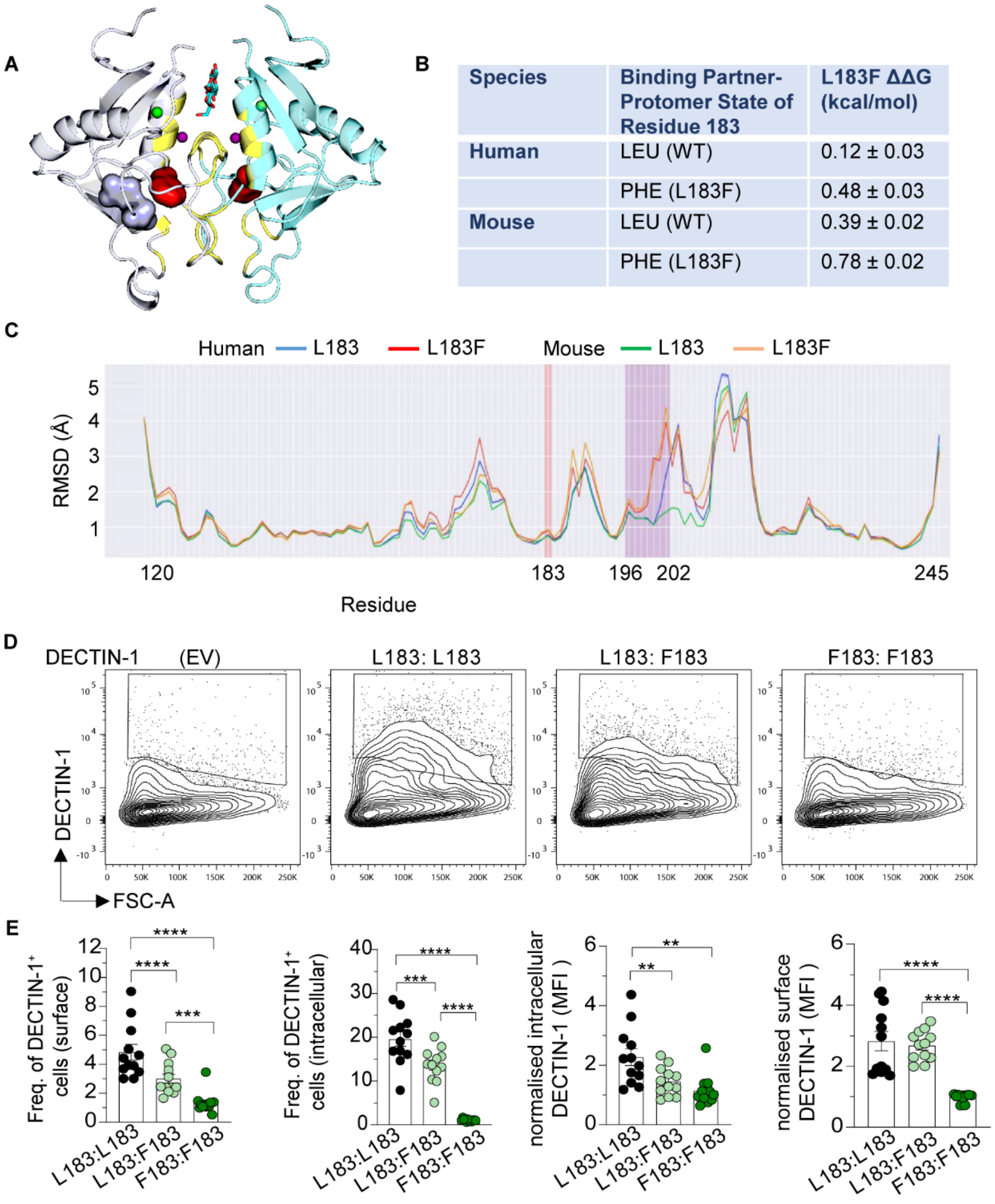

Species	Binding Partner-Protomer State of Residue 183	L183F ΔΔG (kcal/mol)
Human	LEU (WT)	0.12 ± 0.03
	PHE (L183F)	0.48 ± 0.03
Mouse	LEU (WT)	0.39 ± 0.02
	PHE (L183F)	0.78 ± 0.02

Fig. S1. DECTIN-1 molecular dynamic simulations and HEK cell transfections. (A) A visual representation of the reconstructed human dimer interface, including the mutation sites (red), β-glucan ligand (licorice representation) and DECTIN-1 dimer interface (yellow). Green ions are chloride, and magenta ions are calcium. Residues which were involved in the dimer interface but not conserved between mouse and human protein sequences were mutated to generate the reconstructed human dimer/monomer structures and are displayed in silver space-filling representation. **(B)** Free energy values for mouse and reconstructed human dimer MD simulations with healthy/WT (L183) or mutant (L183F) protomer binding partners. **(C)** Root mean square deviation (RMSD) values for mouse and human monomer structures with or without the L183F mutation. The red band region highlights the L/F183 site, while the purple band highlights the F200-containing dimer-forming loop. **(D)** Flow cytometry plots of surface DECTIN-1 expression in HEK293 cells transfected with WT (L183:L183), mutant (F183:F183) and a combination of WT and mutant *CLEC7A* DNA (L183:F183). **(E)** Quantification of surface or intracellular DECTIN-1 expression and MFI values (normalized to empty vector), in transfected HEK293 cells for the indicated transfection conditions. Data is pooled from two independent experiments (n=12, each dot is a separate transfection). Statistical significance in **(E)** was calculated by one-way ANOVA, $^{**}P < 0.01$, $^{***}P < 0.001$ and $^{****}P < 0.0001$.

Fig. S2.

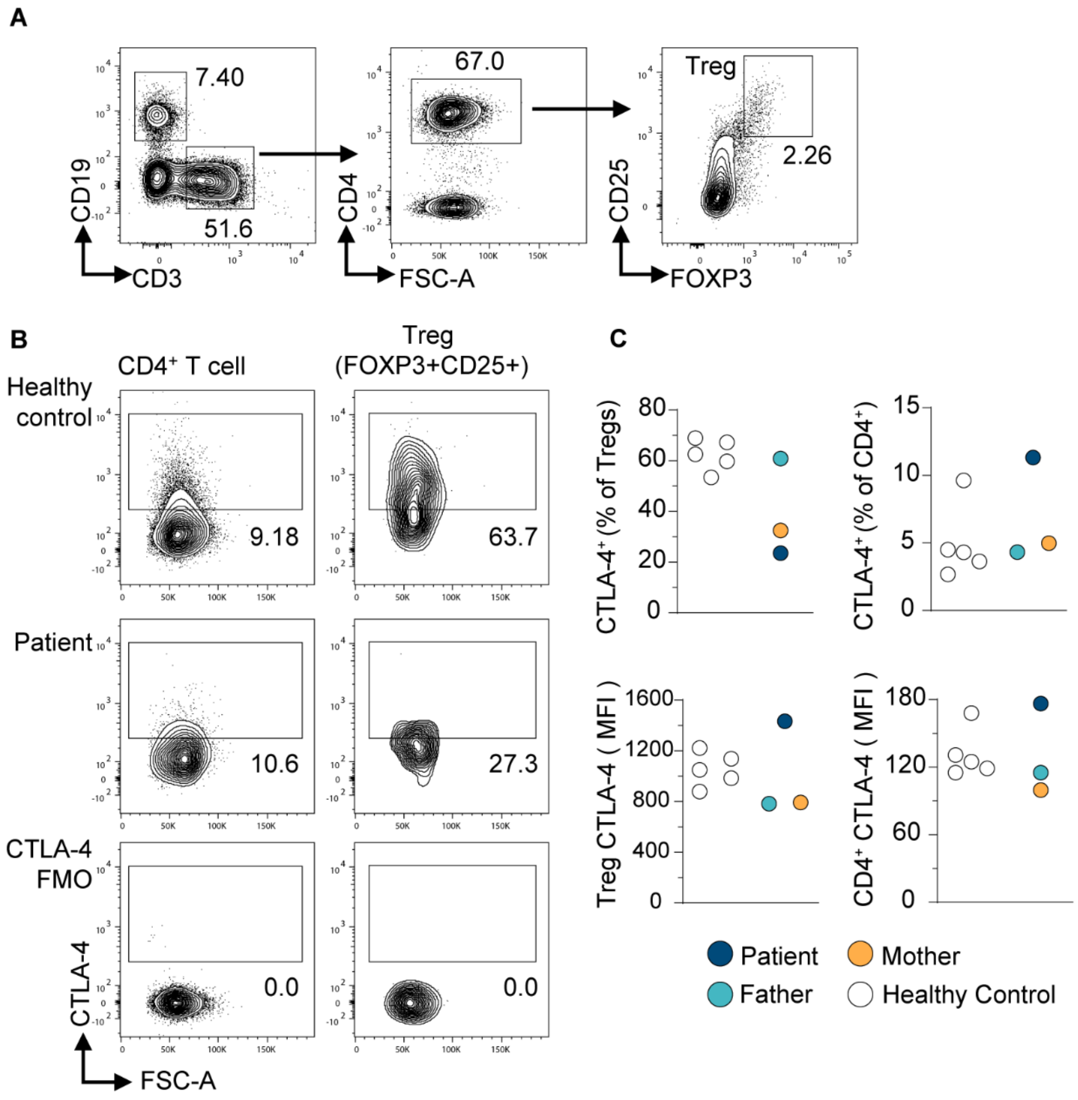

Fig. S2. CTLA-4 expression in kindred samples. (A) Gating strategy for isolating CD4$^+$ T cells and Tregs from live PBMCs. **(B)** Flow cytometric profiles depicting CTLA-4 expression in CD4$^+$ T cells and Treg from a representative healthy control, patient and FMO control. **(C)** Quantification of CTLA-4$^+$ cells and CTLA-4 MFI from CD4$^+$ T cells and Tregs.

Fig. S3.

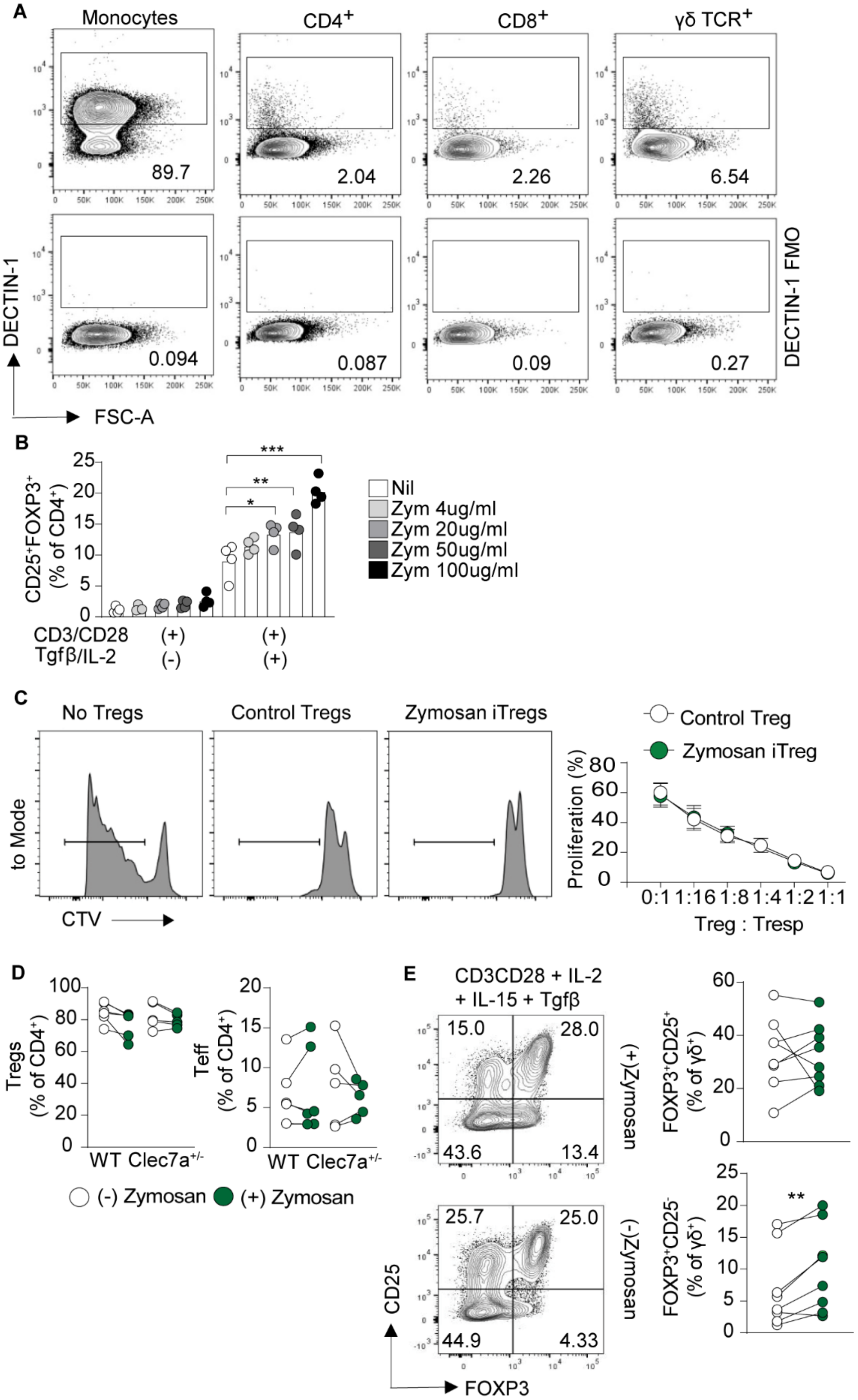

A
Monocytes
CD4+
CD8+
γδ TCR+
89.7
2.04
2.26
6.54
0.094
0.087
0.09
0.27
DECTIN-1 FMO
DECTIN-1
FSC-A
B
CD25+FOXP3+ (% of CD4+)
Nil
Zym 4ug/ml
Zym 20ug/ml
Zym 50ug/ml
Zym 100ug/ml
CD3/CD28 (+) (+)
Tgfβ/IL-2 (-) (+)
C
No Tregs
Control Tregs
Zymosan iTregs
to Mode
CTV
Control Treg
Zymosan iTreg
Proliferation (%)
0:1 1:16 1:8 1:4 1:2 1:1
Treg : Tresp
D
Tregs (% of CD4+)
Teff (% of CD4+)
WT Clec7a+/-
(-) Zymosan (+) Zymosan
E
CD3CD28 + IL-2 + IL-15 + Tgfβ
15.0
28.0
43.6
13.4
25.7
25.0
44.9
4.33
(+)Zymosan
(-)Zymosan
CD25
FOXP3
FOXP3+CD25+ (% of γδ+)
FOXP3+CD25- (% of γδ+)

Fig. S3. Zymosan induced T_{reg} differentiation. (**A**) Representative flow cytometric profiles depicting DECTIN-1 expression in the monocytes and the indicated T cell subsets following CD3/CD28 stimulation with DECTIN-1 FMO controls. **(B)** Dose dependent T_{reg} differentiation with 4, 20, 50 or 100 ug/ml Zymosan, in the absence or presence of rIL-2 (1mg/ml) and rTGF-β (5ng/ml) (n=4, each dot represents average triplicates of a separate healthy blood donor). Human *in vitro* T cell differentiation data is representative of three independent experiments (n > 8), where each dot represents the mean value of cultures set up in triplicate from a single healthy blood donor. (**C**) Flow cytometric plots and quantification showing proliferating $CD4^{+}CD25^{-}$ responder T cells according to dilution of the cytoplasmic fluorescent dye CTV after 3 days of α-CD3 and α-CD28 stimulation in the presence or absence of control or zymosan-induced T_{reg} cells. Data is representative of 2 independent experiments (n=6 per group). **(D)** Quantification of the proportion of T_{regs} ($FOXP3^{+}$) and Teff ($FOXP3^{-}CD44^{+}$) following rTGF-β (2ng/ml) and rIL-2 (5ng/ml) activation during murine T_{reg} differentiation cultures on WT or *Clec7a*$^{+/-}$ naïve $CD4^{+}$ T cells. Data is representative of 2 independent experiments with n = 4-5 per genotype. (**E**) Flow cytometric plots and quantification of conventional ($CD25^{+}$) and unconventional ($CD25^{-}$) γδ T_{regs} ($FOXP3^{+}$) following stimulation with T_{reg} inducing cytokines (rIL-2/rIL-15(50ng/ml)/rTGF-β), with or without zymosan (100ug/ml). Statistical significance in **(B-E)** was calculated by paired t-tests **P < 0.01, ***P < 0.001 and ****P < 0.0001.

Fig. S4.

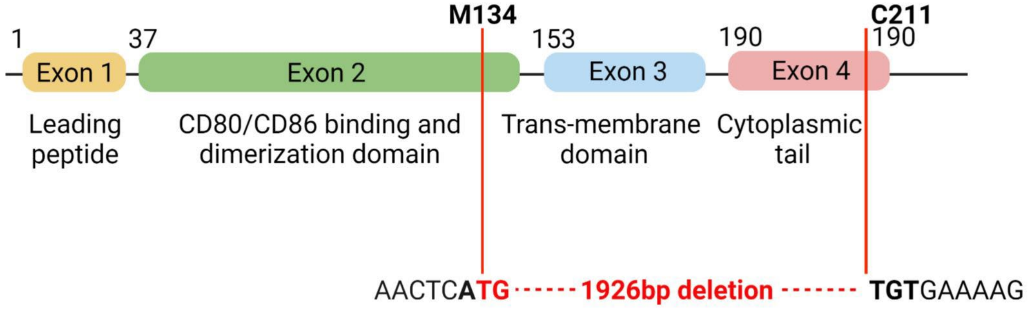

Fig. S4. Generation of the *Ctla4*$^{+/-}$ CRISPR strain. A schematic of the murine Ctla-4 protein domains and encoding exons, with an overlay of the 1926bp (approximately 2kb) deletion in the *Ctla4* gene generation through CRISPR/Cas9. The deletion begins in the "T" encoding M134 in exon 2 which translates into the ligand binding site and dimerization domain and finishes before C211 in exon 4 or the cytoplasmic tail.

Fig. S5.

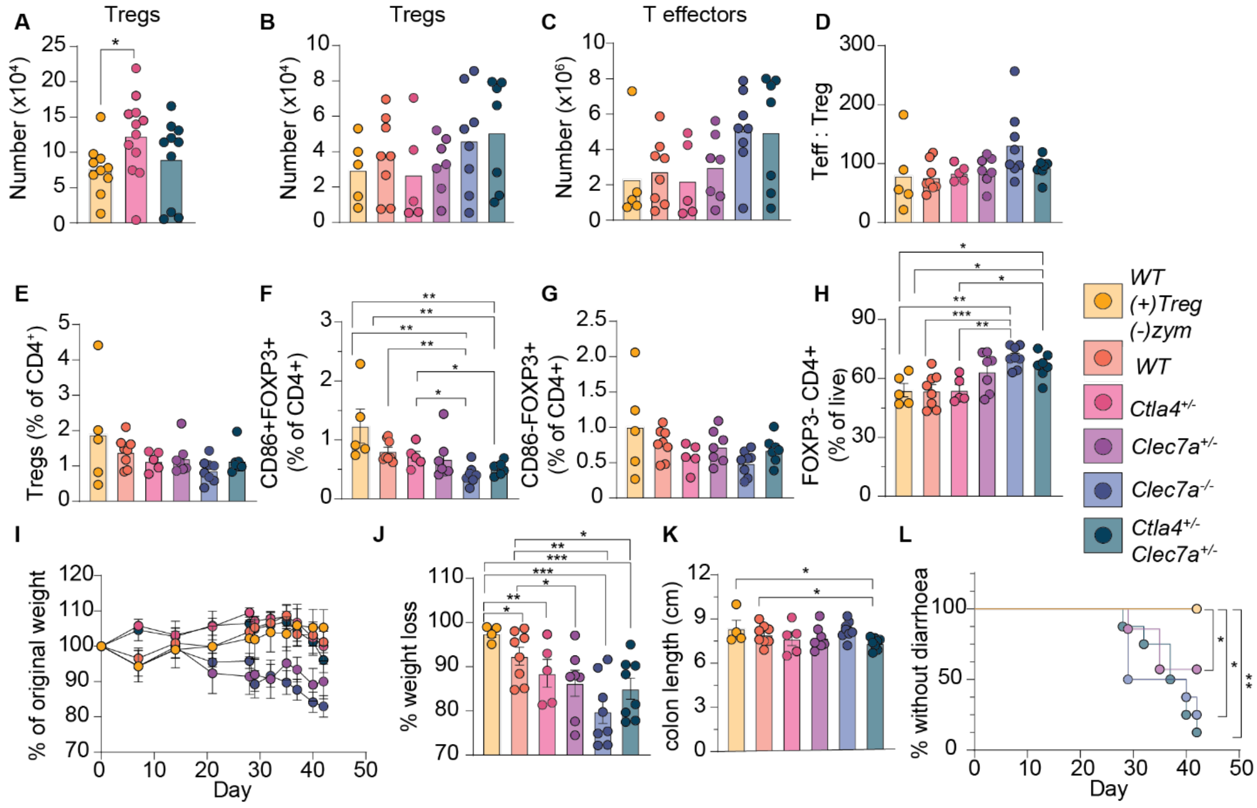

Fig. S5. DECTIN-1 T_{reg} differentiation *in vivo*. (**A**) Absolute splenic Tregs count from WT (white), *Ctla4*$^{+/-}$ (blue) and *Ctla4*$^{+/-}$*Clec7a*$^{+/-}$ (red) mice following 7 days of zymosan (100ug/ml) IP injections. (**B-D**) Absolute colon T_{regs} **(B)** and T effector **(C)** cells counts and Teffector : T_{reg} ratios **(D)** following T cell adoptive transfers. (**E-H**) Frequencies of total T_{regs} (FOXP3^{+}) **(E)**, CD86^{+}T_{regs} (CD86^{+}FOXP3^{+}) **(F)**, CD86^{-}T_{regs} (CD86-FOXP3^{+}) **(G)** and Teffectors (FOXP3^{-} CD4^{+}) **(H)** for the indicated *Rag1*$^{-/-}$ recipients. (**I-J)** Weight loss curves **(I)** and total weight loss frequencies **(J)** for *Rag1*$^{-/-}$ mice over a 42-day monitoring period. (**K**) Colon length for all experimental groups. **(L**) The proportion of *Rag1*$^{-/-}$ mice which did not develop diarrhoea following T cell adaptive transfers with the indicated FOXP3 donor groups. Note the experiment control WT group received both WT T_{regs} and Tnaive cells in the absence of zymosan stimulation, n = 5-12 per genotype. Statistical significance in **(A-H, J-K)** was calculated by non-parametric Mann-Whitney tests, while differences in survival curves **(L)** were analysed with logrank tests *P < 0.05, **P < 0.01 and ***P < 0.001.

Table S1. Routine immunological studies from the patient and his mother.

	Routine immunological studies			
	Pat 19y	Pat 25y	Pat 26y	Mother
IgG g/l	9,07	5,91	5,73	9,99
IgA g/l	0,44	<0.06	<0.06	2,1
IgM g/l	0,62	0,28	0,29	36
IgE IU/ml	0,21			
IgG2 g/l	0,29			3
IgG3 g/l	0,07			0,35
C3 mg/dL		98		107
C4 mg/dL		25.9		23,2
Abs. Lymph x10e3/µl		1,03	1,07	1,97
Abs. Eosinophils x10e3/µl		1,48	0,85	0,66
CD3 %(abs)	66	78,9 (0,81)	76,7 (0,82)	75,8 (1,49)
CD4 %(abs)	41	61,2 (0,63)	57,6 (0,61)	43,2 (0,85)
CD8 %(abs)	17	13,8 (0,14)	14,8 (0,15)	24,7 (0,49)
CD19 %(abs)	10	5.9 (0,06)	5 (0,05)	16,1 (0,32)
Nk %(abs)	24	14,7 (0,15)	17,5 (0,18)	6,7 (0,13)
CD4+CD45Ra+		19,2	23,6	21,9
CD4+CD45Ro+		51,2	51,8	52,1
CD8+CD45Ra+		58,7	60,3	63,8
CD8+CD45Ro+		15,5	10,6	13,4
CD4+CD31+CD45Ra+		22,1	24,3	17,5
TCR ab %		98,9	98,9	92,8
TCR gd %		1	1,1	7
TCRab+CD4-CD8- %		3	3,2	1,7
CD19+IgD+CD27-		82,3	83,7	95
CD19+IgD+CD27+		11,1	9,4	2,4
CD19+IgD-CD27+		2,6	3,6	1,8
CD19+IgMhighCD38high		0,1	4,3	1,5
CD19+CD21low		18,7	17,6	0,9
Th1 %		32,1		32,7
Th2 %		9,8		20,1
Th17 %		13,3		13,2
Tfh %		31,9		18,26
Treg %		6,9		7,1
